# Molecular Mechanism of the β-Lactamase Mediated β-Lactam Antibiotic Resistance of *Pseudomonas aeruginosa* Isolated From a Chinese Teaching Hospital

**DOI:** 10.3389/fmicb.2022.855961

**Published:** 2022-04-28

**Authors:** Hailong Lin, Chunlin Feng, Tingting Zhu, Anqi Li, Shuang Liu, Lei Zhang, Qiaoling Li, Xueya Zhang, Li Lin, Junwan Lu, Xi Lin, Kewei Li, Hailin Zhang, Teng Xu, Changchong Li, Qiyu Bao

**Affiliations:** ^1^Department of Infectious Disease, The Second Affiliated Hospital and Yuying Children’s Hospital, Wenzhou Medical University, Wenzhou, China; ^2^Key Laboratory of Medical Genetics of Zhejiang Province, Key Laboratory of Laboratory Medicine, Ministry of Education, School of Laboratory Medicine and Life Sciences, Wenzhou Medical University, Wenzhou, China; ^3^Department of Pediatric Respiratory Disease, The Second Affiliated Hospital and Yuying Children’s Hospital, Wenzhou Medical University, Wenzhou, China; ^4^Medical Molecular Biology Laboratory, School of Medicine, Jinhua Polytechnic, Jinhua, China; ^5^Institute of Translational Medicine, Baotou Central Hospital, Baotou, China

**Keywords:** *Pseudomonas aeruginosa*, resistance, antimicrobial susceptibility test, β-lactamase gene, pulsed-field gel electrophoresis

## Abstract

*Pseudomonas aeruginosa* can cause infections in the blood, lungs (pneumonia), or other parts of the body after surgery. To investigate the molecular characteristics of β-lactam antibiotic resistance of *P. aeruginosa* isolated from a hospital population between 2015 and 2017, in this study, the antimicrobial susceptibility and the resistance gene profile of the bacteria were determined. The Pulsed-field gel electrophoresis (PFGE) was used to characterize the clonal relatedness and sequencing and comparative genomic analysis were performed to analyze the structure of the resistance gene-related sequences. As a result, of the 260 *P*. *aeruginosa* strains analyzed, the resistance rates for 6 β-lactam antibiotics ranged from 4.6 to 9.6%. A total of 7 genotypes of 44 β-lactamase genes were identified in 23 isolates (8.9%, 23/260). Four transconjugants from different donors carrying *bla*_CARB-3_ exhibited a phenotype of reduced susceptibility to piperacillin–tazobactam, ceftazidime, and cefepime, and 2 transconjugants harboring *bla*_IMP-45_ exhibited a phenotype of reduced susceptibility to carbapenems. *bla*_CARB_ positive isolates (*n* = 12) presented six PFGE patterns, designated groups A to F. Two *bla* genes (*bla*_IMP-45_ and *bla*_OXA-1_) in PA1609 related to a class 1 integron (*intI1*-*bla*_IMP-45-_*bla*_OXA-1_-*aac(6′)-Ib7*-*catB3*-*qacE∆1*-*sul1*) were encoded on a plasmid (pPA1609-475), while the *bla*_CARB-3_ gene of PA1616 also related to a class 1 integron was located on the chromosome. The results suggest that β-lactam antibiotic resistance and clonal dissemination exist in this hospital population. It indicates the necessity for molecular surveillance in tracking β-lactamase-producing strains and emphasizes the need for epidemiological monitoring.

## Introduction

The treatment of *Pseudomonas aeruginosa* (*P*. *aeruginosa*) infection has become a clinical problem because of the emergence of multidrug-resistant bacteria (Subedi et al., 2018). *P*. *aeruginosa* is prevalent in healthcare settings and is commonly found in patients under medical care and surviving on biotic and abiotic surfaces, such as medical equipment (Mohammadpour et al., 2019). It is largely associated with hospital-acquired infections including ventilator-associated pneumonia, central line-associated bloodstream infection, and so on (Philippon et al., 1986; Barnes et al., 2018).

*Pseudomonas aeruginosa* is intrinsically resistant to a variety of antimicrobials, such as β-lactams (Keith, 2011). One of the main drug resistance mechanisms is the production of β-lactamase, an enzyme that hydrolyses β-lactam antibiotics and leads to their inactivation (Livermore and Woodford, 2006). There are many classifications of β-lactamases (Bush and Jacoby, 2010). The β-lactamases classified under the Ambler classification are divided into four classes: Class A ESBLs, Class B MBLs, Class C AmpC, and Class D (OXA type). Two of them (Class A and B) are reported as rapidly developing enzymes in clinical isolates of *P*. *aeruginosa* (Al Dawodeyah et al., 2018).

In *P*. *aeruginosa*, the overexpression of the naturally occurring AmpC is associated with a decreased susceptibility or resistance to expanded spectrum cephalosporins, such as ceftazidime (Mikhail et al., 2019). Plasmid-mediated AmpC was first identified in the 1980s in Gram-negative bacteria (Fallah et al., 2013). However, unlike Enterobacteriaceae, very few reports describe plasmid-borne AmpC in *P*. *aeruginosa*. The sulfhydryl reagent variable (SHV) family of Class A β-lactamases was derived from the chromosome of *Klebsiella* spp., but later incorporated into plasmids (Wolter and Lister, 2013). Variants of SHV have been isolated from *P*. *aeruginosa.* The TEM (Temoneira) type β-lactamases are functionally similar to SHV and were first identified in *Escherichia coli*. Later, many TEM variants were found in *P*. *aeruginosa* with different enzymatic capacities (Livermore and Woodford, 2006; Bush and Jacoby, 2010). Carbapenem resistance in clinical isolates of *A. baumannii* is mediated by overexpression of either OXA-23 or OXA-51 through insertion of IS*Aba1* in their promoter region (Wong et al., 2019). Several variants of OXA enzymes with extended spectrum have been identified in *P*. *aeruginosa* (Shaikh et al., 2015). Other extended-spectrum β-lactamases (ESBL) include PER (penicillins and cephalosporins), GES (penicillins and extended-spectrum cephalosporins), and KPC (carbapenems). Metallo-β-lactamases are unique due to the presence of zinc in their active site. They are plasmid-mediated and highly transferable; however, chromosomal integration is also common. The first metallo-β-lactamase (IMP-1) in *P*. *aeruginosa* was detected in Japan and since then the incidence of these agents has been increasing (Watanabe et al., 1991). Other MGE (mobile genetic elements)-associated metallo-β-lactamase families include SPM, VIM, and GIM (Ghamgosha et al., 2015). These also have been identified in *P*. *aeruginosa* and their prevalence rates are gradually increasing (Queenan and Bush, 2007). Since mutations in the ESBL genes produce enzymes with different specificities for substrates or susceptibilities to β-lactam inhibitors, the numbers of variants are growing.

In this study, the molecular resistance mechanisms of β-lactam antibiotics and the characteristics of the resistance gene-related sequences of 260 clinical *P*. *aeruginosa* isolates were analyzed.

## Materials and Methods

### Bacterial Strains

A total of 260 non-duplicate clinical *P*. *aeruginosa* (hereinafter referred to as PA in strain numbers of *P*. *aeruginosa*) were isolated from an affiliated hospital of Wenzhou Medical University, Zhejiang, China between 2015 and 2017. The strain was identified using the Vitek-60 microorganism auto analysis system (BioMerieux Corporate, Craponne, France). Further verification was performed using homologous comparisons of the sequences of 16S rRNA genes.

### Antimicrobial Susceptibility Test

Antimicrobial susceptibility test was performed using the agar dilution method and interpreted according to the Clinical and Laboratory Standards Institute (CLSI, 2019) guidelines. *P*. *aeruginosa* ATCC 27853 was used as the quality control strain.

### DNA Extraction and Sequencing

Each isolate was incubated overnight in 5 ml of Luria-Bertani (LB) broth (Yamamoto et al., 2021) at 37°C for 16 h, and the genomic DNA was extracted using an AxyPrep Bacterial Genomic DNA Miniprep kit (Axygen Scientific, Union City, CA, United States). For the mixed genomic DNA sequencing, the cultured LB broth of all 260 isolates was mixed and the DNA was extracted. A library with an average insert size of 400 bp was prepared using the NEBNext Ultra II DNA library preparation kit and subsequently sequenced by the Illumina Novaseq (paired-end run; 2 × 150 bp with sequencing depth of about 100X). In addition, for the whole-genome sequencing of a certain bacterium, a 10- to 20-kb insert library was prepared and sequenced (with sequencing depth of about 200X, coverage 100%) by the Pacific Bioscience RSII sequencer at Annoroad Gene Technology Co., Ltd. (Beijing, China).

### Genome Assembly and Annotation

Genome assembly of the mixed DNA sequencing data was performed using megahit.^1^ The complete genome of a *P. aeruginosa* isolate was assembled using Canu^2^ with long reads obtained from PacBio sequencing and the error correction of the tentative complete circular sequence was performed using Pilon^3^ with short read sets of the same isolate derived from Illumina sequencing. Open reading frames (ORFs) of the mixed DNA sequences were predicted using Prodigal^4^ with default parameters. Using the antibiotic resistance genes of the CARD^5^ and ResFinder databases^6^ as a query, a BLASTN search was performed against the assembled sequences of the mixed DNA with thresholds of >70% nucleotide identity and > 80% alignment coverage. Gene prediction and annotation of the genome were initially performed with RAST^7^ and then verified by BLASTP searches against the UniProtKB/Swiss-Prot^8^ and RefSeq databases.^9^ Annotation of mobile genetic elements was carried out using online databases including ISfinder,^10^ INTEGRALL,^11^ and the Tn Number Registry.^12^ Gene organization diagrams were generated using R script^13^ and modified with Inkscape 1.0.^14^

### Screening of the β-Lactamase Resistance Determinants

β-lactamase resistance genes including Amber class A (*bla*_CARB-3_, *bla*_GES-1,_ and *bla*_TEM-1_), class B (*bla*_IMP-45_), class C (*bla*_DHA-1_, *bla*_PDC-3_), and class D (*bla*_OXA-23_, *bla*_OXA-50_, *bla*_OXA-66_) were screened by the polymerase chain reaction (PCR). The primers were designed according to the corresponding publications and synthesized by Shanghai Sunny Biotechnology Co., Ltd. (Shanghai, China; Table 1). The PCR products were verified by sequencing and compared with those in the public database by the BLASTN program.^15^

**Table 1:** Primers used in this work.

Primer	Primer sequence (5′ → 3′)	PCR product size (bp)	Tm (°C)	Restriction endonuclease	References
IMP-45-F	CGGGATCCATGTTTTTGTTTTGTAGCATTACTG	714	60	BamHI	Wang et al., 2014
IMP-45-R	CCAAGCTTTTAATGTGCAGTGGTACTTTTTTTG			HindIII	
GES-1-F	CGGGATCCTTTGTACAGTCTATGCCTCGGG	1,058	60	BamHI	Poirel et al., 2000
GES-1-R	GCTCTAGACGTCGGCTTGAACGAATTGTTA			XbaI	
OXA-23-F	CGGGATCCCATTGAGATGTGTCATAGTATTCGT	1,002	58	BamHI	Sun et al., 2019
OXA-23-R	GCTCTAGATAAAAGGCCCATTTATCTCAAATGG			XbaI	
OXA-1-F	CGGGATCCATTGCAATTTTTTCATGAATTGGCC	1,069	58	BamHI	Culebras et al., 2010
OXA-1-R	CCAAGCTTTGAATACTCCATTTGAACCAGTGGA			HindIII	
OXA-50-F	CGGGATCCGCCTTCTCTTCTTCAGCGCC	711	60	BamHI	Codjoe et al., 2019
OXA-50-R	CCAAGCTTGGGCATGTCGATGTTCAGGG			HindIII	
TEM-1-F	CGGGATCCGACGCTTCATCAGAAGGGCA	1,246	60	BamHI	Rodríguez-Martínez et al., 2009
TEM-1-R	CCAAGCTTCGCCTGGTAAGCAGAGTTTT			HindIII	
CARB-3-F	CGGGATCCATGTGTGACAATCAAAATTATGGGG	895	60	BamHI	Lachapelle et al., 1991
CARB-3-R	CCAAGCTTGCGACTGTGATGTATAAACGTCAAA			HindIII	
PDC-7-F	GCTCTAGATCGAACCATGTCTGCTCCAA	1,420	58	XbaI	Jahan et al., 2020
PDC-7-R	CCAAGCTTAGGGTCATGGCTCCATCATA			HindIII	
DHA-1-F	GCTCTAGAGGTAAAACTGAGATGACGGGC	1,422	58	XbaI	Lee et al., 2011
DHA-1-R	CCAAGCTTCTCATCCTCCATAAAACAGCCC			HindIII	

*Underlined sequences are restriction endonuclease sites*.

### Plasmid Conjugation Experiments

The conjugation experiment of biparental mating on the sterile nitrocellulose filter was performed using the multiple resistant *P. aeruginosa* as the donor cell and the rifampin-resistant *Escherichia coli* C600 (EC600) as the recipient (He et al., 2021). The transconjugants were selected on Mueller–Hinton agar plates containing 600 μg/ml rifampin and 32 μg/ml ceftazidime or 8 μg/ml meropenem. The target resistance gene in the transformant was verified by PCR with primers of the corresponding β-lactamase resistance genes (Table 1). The PCR product was sequenced and the sequence was compared with those in the public database by the BLASTN program (see footnote 15). The plasmid of the transformant was extracted with an AxyPrep Plasmid Miniprep Kit (Axygen Scientific, Union City, CA, United States), and the target resistance gene on the plasmid was also verified by PCR and PCR product sequencing as mentioned above.

### Pulsed-Field Gel Electrophoresis

Pulsed-field gel electrophoresis was performed after digestion of DNA samples with 20 U of *Spe* I restriction enzyme (Takara, Dalian, China). The DNA fragments were separated using a CHEF-Mapper XA PFGE system (Bio-Rad, Hercules, California, United States) for 18 h at 6 V/cm for 14°C, with a pulse angle of 120° and pulse duration of 5 s to 25 s in 1% Seakem Gold agarose with cell suspension buffer (10 ml 1 M Tris–HCl, pH 8.0, 20 ml 0.5 M EDTA, pH 8.0, diluted to 100 ml with sterilized pure water) and cell lysis buffer (25 ml 1 M Tris–HCl, pH 8.0, 50 ml 0.5 M EDTA, pH 8.0, 50 ml 10% Sarcosyl, diluted to 100 ml with sterilized pure water). The restriction patterns were analyzed and interpreted according to the criteria proposed by Tenover, et al. (Bannerman et al., 1995).

### Comparative Genomic Analysis of the β-Lactamase Gene-Related Sequences

For the comparative genomic analysis of the *bla*_CARB-3_, *bla*_IMP45,_ and *bla*_PDC3_ gene-related fragments, sequences containing these *bla* genes were obtained from the NCBI nucleotide database using the *bla*_CARB-3_ gene (S46063.1), the *bla*_IMP45_ gene (NG_049209) or the *bla*_PDC3_ gene (NG_04990) as a query by Blastn searching. The results were retained only if sequences containing a complete *bla*_CARB-3_ (a total of 13 kb with approximately 6 kb both upstream and downstream of the gene), *bla*_IMP45_ (a total of 22 kb with approximately 18 kb upstream and 3 kb downstream of the gene) or *bla*_PDC3_ (a total of 10 kb with approximately 4 kb upstream and 5 kb downstream of the gene). Multiple sequence alignments were performed by MAFFT24^17^ using each of the *bla* gene-related fragments of this work as a reference, and the sequences were clustered and the sequences flanking the resistance gene with an identity of ≥80% was retained. The sequence sharing the highest similarity to the other sequences in each cluster was chosen as the candidate for ortholog analysis. Orthologous groups of the genes from the candidate sequences were identified using BLASTP and InParanoid.^18^ The sequence retrieval, statistical analysis, and other bioinformatics tools used in this study were applied with Python and Biopython scripts.^19^

### Data Availability Statement

The nucleotide sequences of the chromosomes and plasmids of the bacteria of this work have been deposited in GenBank under accession numbers CP090649 (PA1609), CP090650 (pPA1609-475), CP090651 (pPA1609-47), CP090648 (PA1616), and CP090647 (PA1681).

## Results

### Antimicrobial Susceptibility

In this study, the MIC levels of the 12 antibiotics (including oxacillin, piperacillin–tazobactam, cefuroxime, ceftazidime, cefepime, aztreonam, imipenem, meropenem, gentamicin, amikacin, levofloxacin, and polymyxin B) were determined for 260 *P*. *aeruginosa* isolates. A small part of the isolates showed resistance to 6 β-lactams with the resistance rates ranging from 4.6% (piperacillin–tazobactam) to 9.2% (meropenem). They also showed lower resistance rates for other classes of the antibiotics, such as gentamicin (5.4%, 14/260), amikacin (5.4%, 14/260), and levofloxacin (9.2%, 24/260), and the lowest rate of resistance (1.2%, 3/260) for polymyxin B (Table 2).

**Table 2:** Antimicrobial susceptibility of the 260 *P*. *aeruginosa* isolates against 12 antimicrobials tested.

Antimicrobial	*S* (%)	*I* (%)	*R* (%)	MIC50 (μg/ml)	MIC90 (μg/ml)	Range (μg/ml)
Oxacillin				2048	2048	512 ≥ 2048
Piperacillin-Tazobactam	93.08	2.31	4.62	0.5	16	0.5–256
Ceftazidime	88.85	3.85	7.31	4	16	0.5–64
Cefepime	71.92	21.54	6.54	8	16	2–64
Cefuroxime				1,024	2048	128 ≥ 2048
Aztreonam	89.62	3.08	7.31	1	16	0.25–256
Meropenem	51.15	39.62	9.23	2	8	0.0625–64
Imipenem	87.31	3.08	9.62	0.125	8	0.125–64
Polymyxin B	98.85	0	1.15	0.0625	0.25	0.0625–8
Gentamicin	92.69	1.92	5.38	1	2	0.0625- > 128
Amikacin	85.38	9.23	5.38	32	64	8–256
Levofloxacin	89.62	1.15	9.23	0.25	2	0.25–32

### Distribution of β-Lactamase Genes

Among the *bla* genes screened, a total of 44 resistance genes of 7 genotypes from 23 isolates were identified in the 260 *P*. *aeruginosa* isolates with *bla*_OXA_ showing the highest positive rate of 38.6% (17/44). *bla*_CARB_ ranged the second (27.3%, 12/44), and then *bla*_GES-1_ (13.6%, 6/44). The rest 4 (*bla*_TEM_, *bla*_IMP_, *bla*_PDC,_ and *bla*_DHA_) showed lower positive rates of 4.5% (2/44), 4.5% (2/44), 9.1% (4/44) and 2.3% (1/44), respectively. Among the 23 β-lactamase gene-positive strains, more than a half (52.2%, 12/23) carried 2 resistance genes. Three strains carried 3 resistance genes each and one contained 4 resistance genes, while the rest 7 harbored one each (Table 3). The β-lactamase gene harboring isolates showed higher MIC values for the β-lactam antibiotics, especially for the two carbapenem antibiotics (meropenem and imipenem). All (23/23) of these isolates showed MIC levels ≥8 μg/ml for meropenem or imipenem, with 56.5% (13/23) and 60.9% (14/23) showing MIC levels ≥16 μg/ml for cefepime and ceftazidime, respectively (Table 3). In the β-lactamase gene negative strains, there were 11 strains demonstrating resistance to β-lactam antibiotics including 4 strains (1.7%, 4/237) with MIC levels ≥16 μg/ml for meropenem and/or imipenem.

**Table 3:** Antimicrobial susceptibility and β-lactam resistance gene distribution of the 23 β-lactamase gene-positive *P. aeruginosa* isolates.

Isolate	CAZ	FEP	MEM	IMP	AZT	TZP	GM	LEV	PB	Resistance gene			
PA1609	8	16	64	64	64	128	0.0625	0.5	0.25	*bla*_IMP-45_	*bla*_OXA-1_	*bla*_OXA-488_	*bla*_PDC-3_
PA1616	64	64	64	64	64	256	0.125	32	0.25	*bla*_CARB-3_	*bla*_OXA-486_	*bla*_PDC-1_	
PA1642	64	64	64	32	64	128	>128	8	0.25	*bla*_CARB-3_	*bla*_OXA-50_	*bla*_OXA-1_	
PA1646	32	64	64	32	32	64	0.0625	8	0.25	*bla*_CARB-3_	*bla*_OXA-50_	*bla*_OXA-23_	
PA1667	32	32	32	32	128	128	0.0625	8	4	*bla*_CARB-3_	*bla*_OXA-23_		
PA1669	64	64	16	32	8	128	16	16	0.5	*bla*_CARB-3_	*bla*_GES-1_		
PA1680	4	4	64	32	64	32	0.0625	8	0.5	*bla*_OXA-50_	*bla*_OXA-23_		
PA1681	32	32	64	64	32	128	0.0625	8	0.25	*bla*_PDC-3_	*bla*_OXA-50_		
PA1700	16	16	32	32	32	32	0.0625	8	0.25	*bla*_OXA-50_			
PA1701	64	32	32	32	16	128	32	32	0.25	*bla*_CARB-3_	*bla*_OXA-23_		
PA1707	64	32	32	16	16	128	2	32	8	*bla*_CARB-3_	*bla*_GES-1_		
PA1740	32	32	32	16	256	64	0.0625	32	8	*bla*_CARB-3_	*bla*_OXA-23_		
PA1748	4	8	16	16	32	16	0.25	32	0.5	*bla*_GES-1_			
PA1755	32	32	64	64	2	128	0.0625	8	0.5	*bla*_CARB-3_	*bla*_IMP-45_		
PA2358	2	4	16	32	8	8	0.0625	1	0.25	*bla*_GES-1_	*bla*_OXA-50_		
PA2368	2	2	16	16	16	16	2	8	0.5	*bla*_GES-1_			
PA2904	32	32	16	16	32	128	32	32	0.5	*bla*_CARB-3_	*bla*_DHA-1_		
PA2922	4	8	8	16	16	16	0.0625	1	0.5	*bla*_GES-1_			
PA2933	64	64	16	8	8	64	0.0625	0.5	0.25	*bla*_CARB-3_	*bla*_TEM-1_		
PA2939	64	16	32	16	16	32	16	16	0.5	*bla*_OXA-50_	*bla*_TEM-1_		
PA2961	32	32	16	8	16	128	0.125	4	0.5	*bla*_CARB-3_			
PA2972	4	8	64	32	32	16	0.0625	32	0.5	*bla*_OXA-50_			
PA2985	4	4	16	32	16	16	0.0625	2	0.25	*bla*_OXA-50_			
PA1609/EC600	8	8	64	64	4	128	0.0625	0.125	0.125	*bla*_IMP-45_			
PA1701/EC600	32	32	8	8	32	32	0.0625	0.125	0.125	*bla*_CARB-3_			
PA1740/EC600	32	32	8	8	32	32	0.0625	0.125	0.125	*bla*_CARB-3_			
PA1755/EC600	32	32	64	64	2	128	0.0625	0.125	0.125	*bla*_CARB-3_	*bla*_IMP-45_		
PA2933/EC600	32	32	8	8	32	32	0.0625	0.125	0.125	*bla*_CARB-3_			
*E. coli* C600	2	2	1	2	4	8	0.0625	0.125	0.0625				
ATCC 27853	2	2	1	1	2	8	0.0625	0.125	0.0625				

*CAZ, Ceftazidime; FEP, Cefepime; MEM, Meropenem; IMP, Imipenem; AZT, Aztreonam; TZP, Piperacillin-tazobactam; GM, Gentamicin; LEV, Levofloxacin; PB, Polymyxin B*.

### Resistant Plasmid Transferability and Resistance Characteristics

In order to identify the transferability of the β-lactamase genes, we performed conjugation experiments for 5 isolates with high MIC levels to the β-lactam antibiotics. Plasmid detection revealed that they carried one or more large plasmids of more than 100 kb in size. The conjugative plasmids of five strains (PA1609, PA1701, PA1740, PA1755, and PA2933) were successfully transferred to the recipient *Escherichia coli* C600 (EC600). PCR amplification of the β-lactamase genes of the plasmids extracted from the transconjugants revealed that four (PA1701/EC600, PA1740/EC600, PA1755/EC600, and PA2933/EC600) of them carried a *bla*_CARB-3_ gene (including one, PA1755/EC600, co-carrying a *bla*_IMP-45_ gene) and the other one (PA1609/EC600) harbored a *bla*_IMP-45_ gene which was further confirmed by the whole-genome sequencing of the conjugative plasmid of PA1609 (pPA1609-475; Table 4). The 4 transconjugants with *bla*_CARB-3_ exhibited a phenotype of reduced susceptibility to ceftazidime and cefepime (MIC levels of each transconjugant increased up to 16 folds compared to the recipients, respectively), and 2 *bla*_IMP-45_ positive transconjugants exhibited a phenotype of reduced susceptibility to carbapenems (MIC levels increased up to 64 folds compared to the recipient; Table 3).

**Table 4:** Resistance genes and their locations of the sequenced isolates.

Bacterium	PA1609		PA1616		PA1681	
Resistance genes	Chromosome	Plasmid	Chromosome	Plasmid	Chromosome	Plasmid
16S rRNA methyltransferase (G1406)		*armA*				
ABC-F ATP-binding cassette ribosomal protection protein		*msrE*				
aminoglycoside modifying enzyme	*aph(3′)-IIb*		*aph(3′)-IIb*		*aph(3′)-IIb*	
antibiotic efflux pump	*bcr-1*	*qacE*Δ*1*	*bcr-1*		*bcr-1*	
beta-lactamase			*bla*_CARB-3_			
		*bla*_IMP-45_				
		*bla*_OXA-1_				
	*bla*_OXA-488_					
			*bla*_OXA-486_			
					*bla*_OXA-50_	
	*bla*_PDC-3_				*bla*_PDC-3_	
			*bla*_PDC-1_			
Chloramphenicol acetyltransferase	*catB7*		*catB7*		*catB7*	
fosfomycin thiol transferase	*fosA*		*fosA*		*fosA*	
macrolide phosphotransferase		*mphE*				
quinolone resistance protein		*qnrVC1*				
rifampin ADP-ribosyltransferase		*arr-2*				
sulfonamide resistance protein		*sul1*	*sul1*			
trimethoprim resistant dihydrofolate reductase		*dfrA22*				

### Clonal Relatedness of the 12 *bla*_CARB-3_ Carrying *Pseudomonas aeruginosa* Strains

**Figure 1:**
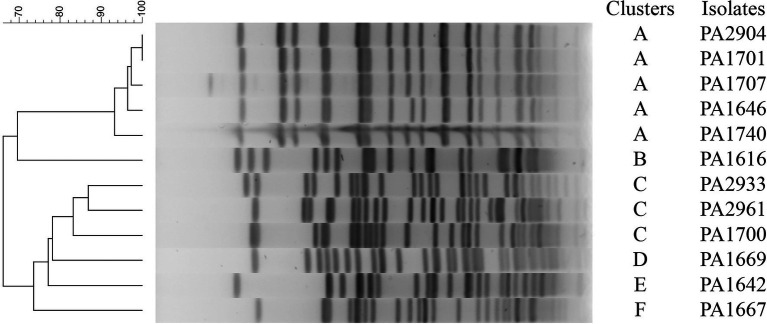
PFGE patterns of 12 *bla*_CARB-3_-positive *Pseudomonas aeruginosa*.

To investigate possible outbreaks of the bacteria carrying the β-lactamase genes, we used PFGE to perform molecular typing for the 12 *bla*_CARB-3_ gene-positive strains. The result showed that the 12 isolates were typable and grouped into 6 clusters designated A to F (Figure 1). The dominant cluster A and B contained 5 and 3 strains, respectively, while the other 4 clusters contained only one strain each.

### General Features of the *Pseudomonas aeruginosa* Genomes

To analyze the molecular structure of the β-lactamase gene-related sequences, the whole-genome sequences of three *P. aeruginosa* isolates carrying different β-lactamase genes with a wider resistance spectrum and higher MIC levels to β-lactam antibiotics were sequenced. It turned out that the three isolates PA1609, PA1616, and PA1681 carried four, three, and two *bla* genes, respectively. All the three carried one *bla*_PDC_ and one or two *bla*_OXA_ genes and an extra *bla* genotype was found in PA1609 (*bla*_IMP_) and PA1616 (*bla*_CARB_), respectively. Besides the β-lactam resistance genes, the three strains also carried several resistance genes of other classes of antibiotics. More resistance genes in the PA1609 genome were encoded on the plasmid. The resistance genes and their locations of the sequenced isolates are shown in Table 4. PA1609 harbored 2 plasmids (a 475 kb plasmid designated pPA1609-475 with *bla*_IMP-45_, *bla*_OXA-1_, *aac(6′)-Ib7,* and so on, and a 47 kb plasmid free of the resistance gene). PA1616 and PA1681 were free of the plasmid.

### Comparative Genomic Analysis of *bla*_IMP-45_, *bla*_CARB-3_, and *bla*_PDC-3_ Related Sequences

**Figure 2:**
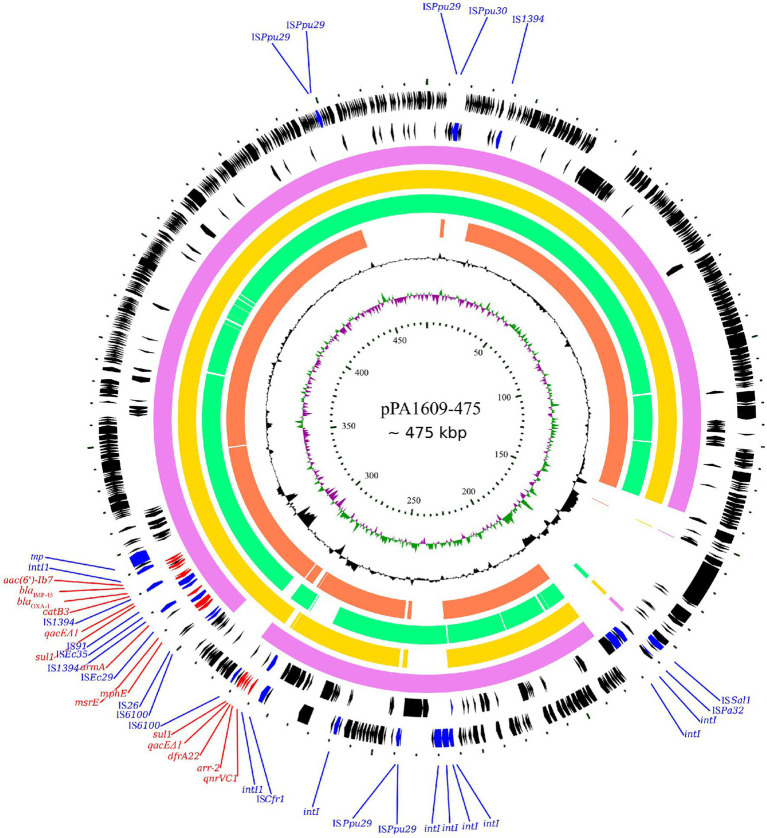
Complete genome sequence of the pPA1609-475 plasmid and comparative genomic analysis of the pPA1609-475 plasmid sequence with other homologous sequences. The circles (from innermost to outermost) represent (i) the scale in kb; (ii) the cumulative GC skew of pPA1609-475; (iii) the GC content of pPA1609-475; (iv) four homologous plasmids pA681-IMP (MF344570.1 from *Pseudomonas aeruginosa*), pPAG5 (CP045003.1 from *P*. *aeruginosa*), pBM413 (CP016215.1 from *P*. *aeruginosa*) and pSY153-MDR (KY883660.1 from P. putida), respectively; (v) the annotated coding sequences of pPA1609-475 with selected genes indicated according to the gene function: antimicrobial resistance genes in red arrows, transposase genes and IS elements in blue arrows.

**Figure 3:**
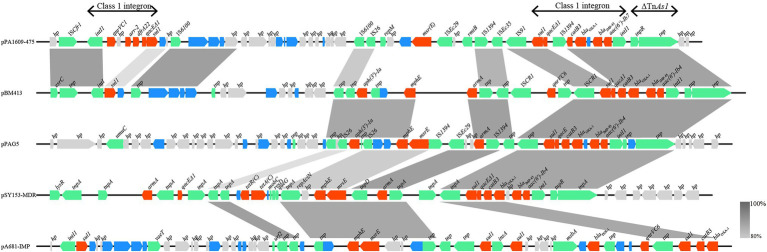
Comparative genomic analysis of the resistance gene regions of pPA1609-475 with other sequences from different bacteria. The homologous gene clusters among the plasmids pPA1609-475, pBM413 (CP016215.1), pPAG5 (CP045003.1), pSY153-MDR (KY883660.1), and pA681-IMP (MF344570.1) with the resistance gene clusters in red. pPA1609-475 contained two class 1 integrons with the *bla*_IMP-45_ encoding one carrying 6 resistance genes (*intI1-bla*_IMP-45_*-bla*_OXA-1_*-aac(6′)-Ib7-catB3-qacE∆1*-*sul1*), and the other one contained 5 resistance genes (*intI1-qnrVC1-aar-2-dfrA22-qacE∆1*-*sul*). Annotated coding sequences are displayed as arrows. Coding sequences are colored based on their assigned gene functions. The genes in blue are those without direct gene names. The grey shading areas indicate homologous regions (identity >80%). hp., hypothetical protein.

Searching the nucleotide sequence data in the NCBI database, four plasmids with the highest percentage of nucleotide sequence similarities with pPA1609-475 from two *Pseudomonas* species, namely, plasmids of pBM413, pPAG5, pSY153-MDR, and pA681-IMP were retrieved (Figure 2). The *bla*_IMP-45_ encoding plasmid pPA1609-475 harbored a total of 14 resistance genes which clustered in two regions with region A of 25 kb in length (position 265–290 kb) and region B of 25 kb in length (position 310–335 kb). These resistance genes were related to the mobile genetic elements including two class 1 integrons and several transposons or insertion sequences. One class 1 integron which contained *bla*_IMP-45_ encoded 6 resistance genes (265–290 kb), and the other one contained 5 resistance genes (310–335 kb; Figures 1, 3).

**Figure 4:**
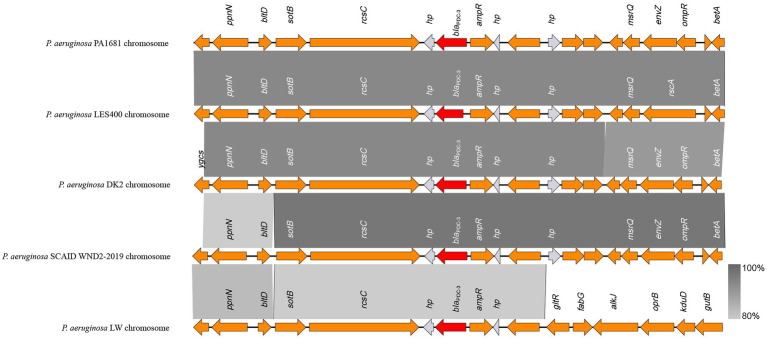
Comparative genomic analysis of the *bla*_PDC-3_-encoding fragment of PA1681 with other sequences from *Pseudomonas aeruginosa* strains: *P. aeruginosa* LES400 (CP006982.1), *P. aeruginosa* DK2 (CP003149.1), *P. aeruginosa* SCAID WND2-2019 (CP041786.1), and *P. aeruginosa* LW (CP022478.1). Annotated coding sequences are displayed as arrows. The grey shading areas indicate homologous regions (identity >80%). hp., hypothetical protein.

**Figure 5:**
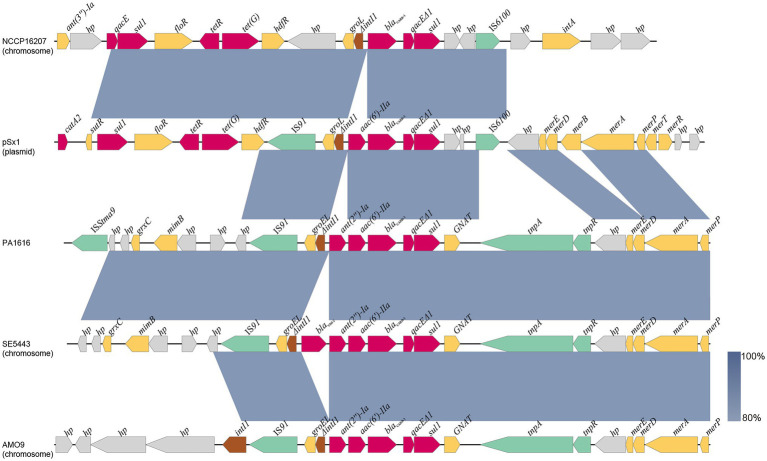
Comparative analysis of the *bla*_CARB-3_ of PA1616 encoding region with other sequences from different bacteria: *Achromobacter xylosoxidans* AMO9 (JX448550.1), *P. aeruginosa* SE5443 (CP046405.1), *Salmonella enterica* NCCP16207 (CP041976.1), and *Shewanella xiamenensis* plasmid pSx1 (CP013115.1). The *bla*_CARB-3_ genes were all related to class 1 integrons. Coding sequences are colored based on their assigned gene functions. The blue shading areas indicate homologous regions (identity >80%). hp., hypothetical protein.

As mentioned above, the *bla*_CARB-3_ of *P*. *aeruginosa* PA1616 and *bla*_PDC-3_ of *P*. *aeruginosa* PA1681 were in the chromosomes. The *bla*_CARB-3_ gene was related to a class 1 integron. The comparative genomic analysis of the area of about 65 kb in length with *bla*_CARB-3_ carrying integron at the center showed that only two other sequence fragments carrying the integrons and with the sequence identity 100% and coverage ≥96% to that of PA1616 were available in the NCBI nucleotide database. The sequence of the clone fosmid AMO9 of *Achromobacter xylosoxidans* X02736 (JX448550.1) contained the same integron structure as that of *P*. *aeruginosa* PA1616, while the integron encoded in the sequence of *P*. *aeruginosa* strain SE5443 chromosome (CP046405.1) contained a VIM-2 gene inserted between *grol* and *ant(2”)Ia* (Figures 4, 5).

## Discussion

In this study, a total of 7 *bla* genotypes of all Amber classes including classes A, B, C, and D genes were identified in 23 of 260 clinical *P*. *aeruginosa* isolates, of which *bla*_CARB-3_ was the most prevalent with 4.6% (12/260) of the isolates carrying it. The PFGE data indicated that all 12 *P*. *aeruginosa* strains bearing *bla*_CARB-3_ in this study were divided into 6 groups. Except two groups included 5 and 3 strains, respectively, the other four groups contain only one isolate each. This result not only demonstrated the distribution of the *bla*_CARB-3_ gene over the pathogens of different genetic backgrounds but also the potential epidemic threat of *bla*_CARB-3_ positive *P*. *aeruginosa* isolates over the hospital populations.

In this study, two strains (0.8%, 2/260) carrying *bla*_IMP45_ showed higher MIC levels to meropenem and imipenem (both 64 μg/ml). As less frequently, but much powerful carbapenem-hydrolyzing enzyme in *P*. *aeruginosa*, IMP45 belongs to the class B β-lactamase (a metallo-β-lactamase, MBLs). On the other hand, seven strains carrying *bla*_GES-1_ showed weak carbapenem resistance. *bla*_GES-1_ was first found in a *Klebsiella pneumoniae* strain isolated from a patient hospitalized in French Guiana (GES stands for Guiana ESBL) in 1998, which has been identified in *Enterobacteriaceae* (*K*. *pneumoniae*, *Enterobacter cloacae*, or *Escherichia coli*) and *P*. *aeruginosa*. The other variant of the GES has been found to have strong hydrolyzing activities against the β-lactams including *bla*_GES-2_, *bla*_GES-5_, *bla*_GES-24_, and *bla*_GES-11_, especially *bla*_GES-5_ (Liu et al., 2018b; Xu et al., 2018).

Besides 12 isolates with 2 β-lactamase genotypes, 4 strains carried three or four different β-lactamase genotypes or subgenotypes of *bla*_CARB_, *bla*_OXA_*, bla*_IMP,_ and *bla*_PDC_ (all isolates containing 1 or 2 *bla*_OXA_ genes, 3 with a *bla*_CARB_ gene, 2 with a *bla*_PDC_ gene, and 1 carrying a *bla*_IMP-45_ gene). Until recently, no similar drug resistance gene combinations, particularly co-occurrence with *bla*_CARB-3_ were reported. These strains showed a wider resistance spectrum and higher resistance levels to the β-lactam antibiotics tested in this study. Co-occurrence of β-lactamase genes displays high rates of resistance to broad-spectrum cephalosporins owing to induced expression of *ampC* β-lactamase gene, and an increasing number of organisms harboring plasmid-encoded extended-spectrum b-lactamase (ESBL) or/and carbapenemase genes have been described (Jahan et al., 2020). Co-occurrence of β-lactamase genes has been reported worldwide. The most common is the co-occurrence of two different β-lactamase genes, in particular, the combination of two different *bla*OXA variants is very common. Co-occurrence of three or more β-lactamase genes is very rare. The emergence of multidrug-resistant *K*. *pneumoniae* isolates, which produces *bla*_VIM-4_, *bla*_CTX-M-15_, *bla*_TEM-1_, *bla*_CMY-4_ have been reported from France (Ktari et al., 2006). Co-occurrence of *bla_NDM-1_* with *bla_OXA-23_* or *bla_OXA-58_* in clinical multidrug-resistant *Acinetobacter baumannii* isolates was also found in Algeria (Ramoul et al., 2016).

The whole-genome sequencing of three isolates (PA1609, PA1616, and PA1681) demonstrated that most of the resistance genes were related to the mobile genetic elements and might be located on the plasmids. The resistance genes of pTL1609-475 (an IncFII-like plasmid) were mainly clustered in two MDR (multidrug-resistant) regions, while in the other plasmids with the most similarity with that of pTL1609-475, only one MDR region was identified. The MDR region of PA1609 encoding a class 1 integron which carried both *bla*_IMP-45_ and *bla*_OXA-1_ were similar to those on the plasmid pBM413 (Liu et al., 2018a), pPAG5, and pSY153. It has been reported that the IncFII-like plasmid had superiority to capture *bla*_IMP45_ by mobile gene elements, resulting in gradual acquisition or accumulation of carbapenem resistance in ST847 *E. coli* (Wang et al., 2020). Compared with pBM413 and other plasmids with similar MDR regions, MDR of pTL1609-475 had undergone much more massive insertions of foreign resistance genetic contents and showed a higher degree of genomic plasticity.

The *bla*_PDC_ genes of PA1616 and PA1681 were located in the chromosomes, of which the one of PA1681 was carried by a class 1 integron, while the other in PA1616 was related to a Tn*As2* transposon. Tn*As2*, a derivative of Tn*3*, was first identified in *Aeromonas salmonicida* (Zamora-Lagos et al., 2015), and then *Escherichia coli*, which has also been reported to carry other resistance genes, such as *aacA4* and *qnrVC6* in addition to *bla*_IMP45_ in a region derived from a class 1 integron. More interestingly, we identified the complete structure of IntI1 coupled with Tn*As2* transposon in plasmid genomes rather than only the IntI1-derived ARGs. Our results indicated the high possibility of horizontal transmission of ARGs by IntI1 through plasmids. In addition, compared with SE5443, an IS*Stma9* element adjacent to *bla*_CARB-3_ was identified in PA1616, which might indicate the high possibility of horizontal transmission of ARGs by IntI1 from different DNA molecules.

## Conclusion

In this work, among the 23 strains that were positive for the β-lactamase gene, most carried two or more β-lactam resistance genes. The *bla*_CARB-3_ gene is the most common β-lactam resistance gene in those isolates and some *bla*_CARB-3_ positive isolates presented clonal relatedness. The β-lactamase gene was related to the mobile genetic elements, such as class 1 integron, located both on plasmids and chromosomes. The β-lactamase gene carrying plasmid was transferable and the transconjugant with *bla*_CARB-3_ exhibited the phenotype of reduced susceptibility to piperacillin–tazobactam, ceftazidime, and cefepime, while the two co-carrying *bla*_IMP-45_ also exhibited reduced susceptibility to carbapenems. These results suggest that resistance to β-lactam antibiotics and clonal dissemination is prevalent in the hospital population of *P. aeruginosa*.

## Data Availability Statement

The datasets presented in this study can be found in online repositories. The names of the repository/repositories and accession number(s) can be found in the article/supplementary material.

## Ethics Statement

Individual patient data were not involved, and only anonymous clinical residual samples during routine hospital laboratory procedures were used in this study. This study followed the principles stated in the Declaration of Helsinki (https://www.wma.net/policies-post/wma-declaration-of-helsinki-ethical-principles-for-medical-research-involving-human-subjects/) and was approved by the Second Affiliated Hospital and Yuying Children’s Hospital of Wenzhou Medical University.

## Author Contributions

TX, CL, and QB designed the study. HL, CF, TZ, QL, and XZ acquired the data. HL, CF, AL, SL, LZ, LL, and JL performed the results analysis and interpreted the data. HL and QB wrote the first draft of the paper. KL, HZ, and TX revised it critically for important intellectual content. All authors contributed to the article and approved the submitted version.

## Funding

This study was supported by the Science & Technology Project of Wenzhou City, China (N20210001 and Y20210003), Zhejiang Provincial Natural Science Foundation of China (LY19C060002 and LQ17H190001), and the National Natural Science Foundation of China (81973382, 81960381, and 81700011).

## Conflict of Interest

The authors declare that the research was conducted in the absence of any commercial or financial relationships that could be construed as a potential conflict of interest.

## Publisher’s Note

All claims expressed in this article are solely those of the authors and do not necessarily represent those of their affiliated organizations, or those of the publisher, the editors and the reviewers. Any product that may be evaluated in this article, or claim that may be made by its manufacturer, is not guaranteed or endorsed by the publisher.

## Footnotes

^1^ https://github.com/voutcn/megahit

^2^ https://github.com/marbl/canu

^3^ https://github.com/broadinstitute/pilon

^4^ https://github.com/hyattpd/Prodigal

^5^ http://arpcard.mcmaster.ca

^6^ https://cge.cbs.dtu.dk/services/ResFinder/

^7^ http://rast.nmpdr.org/

^8^ https://www.expasy.org/resources/uniprotkb-swiss-prot

^9^ https://www.ncbi.nlm.nih.gov/refseq/

^10^ https://github.com/thanhleviet/ISfinder-sequences

^11^ http://integrall.bio.ua.pt/

^12^ http://transposon.lstmed.ac.uk/

^13^ https://www.r-project.org/

^14^ https://inkscape.org/en/

^15^ http://blast.ncbi.nlm.nih.gov/Blast.cgi

^17^ https://mafft.cbrc.jp/alignment/software/

^18^ https://inparanoid.sbc.su.se/

^19^ https://pypi.python.org/pypi/biopython

## References

[ref1] Al Dawodeyah H. Y. Obeidat N. Abu-Qatouseh L. F. Shehabi A. A. (2018). Antimicrobial resistance and putative virulence genes of *Pseudomonas aeruginosa* isolates from patients with respiratory tract infection. GERMS 8, 31–40. doi: 10.18683/germs.2018.1130, PMID: 29564246 PMC5845973

[ref2] Bannerman T. L. Hancock G. A. Tenover F. C. Miller J. M. (1995). Pulsed-field gel electrophoresis as a replacement for bacteriophage typing of *Staphylococcus aureus*. J. Clin. Microbiol. 33, 551–555. doi: 10.1128/JCM.33.3.551-555.1995, PMID: 7751356 PMC227989

[ref3] Barnes M. D. Bethel C. R. Alsop J. Becka S. A. Rutter J. D. Papp-Wallace K. M.. (2018). Inactivation of the *pseudomonas*-derived *Cephalosporinase-3* (*PDC-3*) by relebactam. Antimicrob. Agents Chemother. 62, e02406–e02417. doi: 10.1128/AAC.02406-17, PMID: 29530851 PMC5923161

[ref4] Bush K. Jacoby G. A. (2010). Updated functional classification of β-lactamases. Antimicrob. Agents Chemother. 54, 969–976. doi: 10.1128/AAC.01009-09, PMID: 19995920 PMC2825993

[ref5] Codjoe F. S. Brown C. A. Smith T. J. Miller K. Donkor E. S. (2019). Genetic relatedness in carbapenem-resistant isolates from clinical specimens in Ghana using ERIC-PCR technique. PLoS One 14:e0222168. doi: 10.1371/journal.pone.0222168, PMID: 31513633 PMC6742460

[ref6] Culebras E. González-Romo F. Head J. Gómez M. Morales G. Picazo J. J. (2010). Outbreak of *Acinetobacter baumannii* producing OXA-66 in a Spanish hospital: epidemiology and study of patient movements. Microb. Drug Resist. 16, 309–315. doi: 10.1089/mdr.2009.0113, PMID: 20528099

[ref902] Fallah F. Borhan R. S. Hashemi A. (2013). Detection of bla (IMP) and bla (VIM) metallo-β-lactamases genes among *Pseudomonas aeruginosa* strains. Int. J. Burns Trauma 3, 122–124., PMID: 23638331 PMC3636667

[ref7] Ghamgosha M. Shahrekizahedani S. Kafilzadeh F. Bameri Z. Taheri R. A. Farnoosh G. (2015). Metallo-β-lactamase VIM-1, SPM-1, and IMP-1 genes among clinical *Pseudomonas aeruginosa* species isolated in Zahedan. Iran. Jundishapur J. Microbiol. 8:e17489. doi: 10.5812/jjm.8(4)2015.17489, PMID: 26034547 PMC4449845

[ref8] He J. Sun L. Zhang L. Leptihn S. Yu Y. Hua X. (2021). A Novel SXT/R391 Integrative and Conjugative Element Carries Two Copies of theblaNDM-1Gene in Proteus mirabilis. mSphere. 6:e0058821. doi: 10.1128/mSphere.00588-21, PMID: 34378988 PMC8386438

[ref9] Jahan M. I. Rahaman M. M. Hossain M. A. Sultana M. (2020). Occurrence of intI1-associated VIM-5 carbapenemase and co-existence of all four classes of β-lactamase in carbapenem-resistant clinical *Pseudomonas aeruginosa* DMC-27b. J. Antimicrob. Chemother. 75, 86–91. doi: 10.1093/jac/dkz426, PMID: 31647552

[ref10] Keith P. (2011). *Pseudomonas aeruginosa*: resistance to the max. Front. Microbiol. 2:65. doi: 10.3389/fmicb.2011.00065, PMID: 21747788 PMC3128976

[ref11] Ktari S. Arlet G. Mnif B. Gautier V. Mahjoubi F. Ben Jmeaa M.. (2006). Emergence of multidrug-resistant *Klebsiella pneumoniae* isolates producing *VIM-4* metallo-β-lactamase, CTX-M-15 extended-spectrum β-lactamase, and CMY-4 AmpC β-lactamase in a Tunisian university hospital. Antimicrob. Agents Chemother. 50, 4198–4201. doi: 10.1128/AAC.00663-06, PMID: 17015633 PMC1694011

[ref12] Lachapelle J. Dufresne J. Levesque R. C. (1991). Characterization of the blaCARB-3 gene encoding the carbenicillinase-3 beta-lactamase of *Pseudomonas aeruginosa*. Gene 102, 7–12. doi: 10.1016/0378-1119(91)90530-O, PMID: 1650733

[ref13] Lee C.-H. Liu J.-W. Li C.-C. Chien C.-C. Tang Y.-F. Su L.-H. (2011). Spread of ISCR1 elements containing blaDHA-1 and multiple antimicrobial resistance genes leading to increase of flomoxef resistance in extended-Spectrum-β-lactamase-producing *Klebsiella pneumoniae*. Antimicrob. Agents Chemother. 55, 4058–4063. doi: 10.1128/AAC.00259-11, PMID: 21746945 PMC3165323

[ref14] Liu J. Li L. Peters B. M. Li B. Chen D. Xu Z.. (2018a). Complete genomic analysis of multidrug-resistance *Pseudomonas aeruginosa* Guangzhou-Pae617, the host of megaplasmid pBM413. Microb. Pathog. 117, 265–269. doi: 10.1016/j.micpath.2018.02.049, PMID: 29486277

[ref15] Liu J. Yang L. Chen D. Peters B. M. Li L. Li B. (2018b). Complete sequence of pBM413, a novel multidrug resistance megaplasmid carrying qnr*VC6* and Bla *IMP-45* from *Pseudomonas aeruginosa*. Int. J. Antimicrob. Agents 51, 145–150. doi: 10.1016/j.ijantimicag.2017.09.008, PMID: 28923459

[ref16] Livermore D. M. Woodford N. (2006). The β-lactamase threat in *Enterobacteriaceae*, *pseudomonas* and *Acinetobacter*. Trends Microbiol. 14, 413–420. doi: 10.1016/j.tim.2006.07.008 16876996

[ref17] Mikhail S. Singh N. B. Kebriaei R. Rice S. A. Stamper K. C. Castanheira M.. (2019). Evaluation of the synergy of Ceftazidime-Avibactam in combination with Meropenem, Amikacin, Aztreonam, Colistin, or Fosfomycin against well-characterized multidrug-resistant *Klebsiella pneumoniae* and *Pseudomonas aeruginosa*. Antimicrob. Agents Chemother. 63, e00779–e00719. doi: 10.1128/AAC.00779-19, PMID: 31182535 PMC6658738

[ref18] Mohammadpour B. Rouhi S. Moradi M. Ramazanzadeh R. Saniyi E. Zandi S.. (2019). Prevalence of metallo-β-lactamases in *Acinetobacter baumannii* in Iran: A review and meta-analysis. Infect. Disord. Drug Targets 19, 350–361. doi: 10.2174/1871526518666181016101430, PMID: 30324896

[ref901] Philippon A. M. Paul G. C. Thabaut A. P. Jacoby G. A. (1986). Properties of a novel carbenicillin-hydrolyzing β-lactamase (CARB-4) specified by an IncP-2 plasmid from *Pseudomonas aeruginosa*. Antimicrob. Agents Chemother. 29, 519–520. doi: 10.1128/AAC.29.3.519, PMID: 3087285 PMC180426

[ref19] Poirel L. Le Thomas I. Naas T. Karim A. Jacoby P. (2000). Biochemical sequence analyses of GES-1, a novel class A extended-Spectrum β-lactamase, and the class 1 integron In52 from *Klebsiella pneumoniae*. Antimicrob. Agents Chemother. 44, 622–632. doi: 10.1128/AAC.44.3.622-632.2000, PMID: 10681329 PMC89737

[ref20] Queenan A. M. Bush K. (2007). Carbapenemases: the versatile β-lactamases. Clin. Microbiol. Rev. 20, 440–458. doi: 10.1128/CMR.00001-07, PMID: 17630334 PMC1932750

[ref21] Ramoul A. Loucif L. Bakour S. Rolain J. M. Dekhil M. Rolain J. M. (2016). Co-occurrence of bla*NDM-1* with bla*OXA-23* or bla*OXA-58* in clinical multidrug-resistant *Acinetobacter baumannii* isolates in Algeria. J. Glob. Antimicrob. Resist. 6, 136–141. doi: 10.1016/j.jgar.2016.05.003, PMID: 27530856

[ref22] Rodríguez-Martínez J. M. Poirel L. Nordmann P. (2009). Extended-spectrum cephalosporinases in *Pseudomonas aeruginosa*. Antimicrob. Agents Chemother. 53, 1766–1771. doi: 10.1128/AAC.01410-08, PMID: 19258272 PMC2681535

[ref23] Shaikh S. Fatima J. Shakil S. Rizvi S. M. Kamal M. A. (2015). Antibiotic resistance and extended spectrum β-lactamases: types, epidemiology and treatment. Saudi J. Biol. Sci. 22, 90–101. doi: 10.1016/j.sjbs.2014.08.002, PMID: 25561890 PMC4281622

[ref24] Subedi D. Vijay A. K. Willcox M. (2018). Overview of mechanisms of antibiotic resistance in *Pseudomonas aeruginosa*: an ocular perspective. Clin. Exp. Optom. 101, 162–171. doi: 10.1111/cxo.12621, PMID: 29044738

[ref25] Sun H. Xiao G. Zhang J. Pan Z. Chen Y. Xiong F. (2019). Rapid simultaneous detection of Bla (oxa-23), Ade-B, int-1, and ISCR-1 in multidrug-resistant *Acinetobacter baumannii* using single-tube multiplex PCR and high resolution melting assay. Infect. Drug Resist. 12, 1573–1581. doi: 10.2147/IDR.S207225, PMID: 31289445 PMC6565808

[ref26] Wang Y. Wang X. Schwarz S. Zhang R. Lei L. Liu X.. (2014). IMP-45-producing multidrug-resistant *Pseudomonas aeruginosa* of canine origin. J. Antimicrob. Chemother. 69, 2579–2581. doi: 10.1093/jac/dku133, PMID: 24777897

[ref27] Wang M. Zeng Z. Jiang F. Zheng Y. Shen H. Macedo N.. (2020). Role of enterotoxigenic *Escherichia coli* prophage in spreading antibiotic resistance in a porcine-derived environment. Environ. Microbiol. 22, 4974–4984. doi: 10.1111/1462-2920.15084, PMID: 32419209

[ref28] Watanabe M. Iyobe S. Inoue M. Mitsuhashi S. (1991). Transferable imipenem resistance in *Pseudomonas aeruginosa*. Antimicrob. Agents Chemother. 35, 147–151. doi: 10.1128/AAC.35.1.147, PMID: 1901695 PMC244956

[ref29] Wolter D. J. Lister P. D. (2013). Mechanisms of β-lactam resistance among *Pseudomonas aeruginosa*. Curr. Pharm. Des. 19, 209–222. doi: 10.2174/138161213804070311 22894618

[ref30] Wong M. H.-Y. Chan B. K.-W. Chan E. W.-C. Chen S. (2019). Over-expression of ISAba1-linked intrinsic and exogenously acquired OXA type Carbapenem-hydrolyzing-class D-ß-lactamase-encoding genes is key mechanism underlying Carbapenem resistance in Acinetobacter baumannii. Front. Microbiol. 10:2809. doi: 10.3389/fmicb.2019.02809, PMID: 31866977 PMC6904305

[ref31] Xu T. Wang J. Ying J. Zhu T. Liu Y. Xu L.. (2018). Characterisation of a class 1 integron associated with the formation of quadruple bla *GES-5* cassettes from an IncP-1β group plasmid in *Pseudomonas aeruginosa*. Int. J. Antimicrob. Agents 52, 485–491. doi: 10.1016/j.ijantimicag.2018.07.002, PMID: 30012438

[ref32] Yamamoto K. Toya S. Sabidi S. Hoshiko Y. Maeda T. (2021). Diluted Luria-Bertani medium vs. sewage sludge as growth media: comparison of community structure and diversity in the culturable bacteria. Appl. Microbiol. Biotechnol. 105, 3787–3798. doi: 10.1007/s00253-021-11248-4, PMID: 33856534

[ref33] Zamora-Lagos M. A. Pfeiffer F. Habermann B. Gruber S. (2015). Blettinger M. ISfinder. Available at: https://www-is.biotoul.fr/scripts/ficheIS.php?name=TnAs2 (Accessed July 30, 2020).

